# Editorial: Hereditary Breast and Ovarian Cancer: Current Concepts of Prevention and Treatment

**DOI:** 10.3389/fonc.2020.618369

**Published:** 2020-12-02

**Authors:** Anne Grabenstetter, Conxi Lazaro, Gulisa Turashvili

**Affiliations:** ^1^Department of Pathology, Memorial Sloan Kettering Cancer Center, New York, NY, United States; ^2^Hereditary Cancer Program, Catalan Institute of Oncology, Barcelona, Spain; ^3^Department of Laboratory Medicine and Pathobiology, University of Toronto, Mount Sinai Hospital, Toronto, ON, Canada

**Keywords:** breast, ovary, hereditary, prevention, treatment

Breast cancer is the leading cause of cancer-related death in women worldwide. Whilst ovarian cancer is less common, it remains challenging due to late detection and high mortality (1). Most cases are considered sporadic; however, both tumor types may occur in patients with inherited mutations in cancer susceptibility genes (2). Hereditary breast and ovarian cancer syndrome (HBOC) accounts for 90% of the hereditary neoplasms and is predominantly associated with germline mutations in *BRCA1* or *BRCA2* genes (3). The mean cumulative risk of breast cancer is 57% in *BRCA1* mutation carriers and 49% in *BRCA2* mutation carriers, while ovarian cancer risk in women with *BRCA1* and *BRCA2* mutations is 40% and 18%, respectively. HBOC can also increase the risk, albeit to a lesser extent, for other neoplasms such as prostate or pancreatic cancer and malignant melanoma (3). Since the discovery of the *BRCA* genes and the development of clinical testing, the health advantages of identifying individuals at risk for HBOC have been well documented leading to the investigation and implementation of genetic counseling and screening, enhanced surveillance as well as surgical and non-surgical risk reduction options. We hope that a collection of review (including systematic review), mini-review, perspective and original research articles in this Research Topic will provide further insight on HBOC, including clinicopathologic features of associated cancers, genetic testing and treatment modalities, and their impact on patient outcomes.

The most common and well-characterized genes implicated in HBOC are *BRCA1* and *BRCA2*. Hatano et al. provide a comprehensive summary of the molecular biology of these genes, with a brief history of their discovery and review of the clinical implications of mutations (4). They also discuss the emerging research into the concept of “mutational signatures,” representing the characteristic combination of mutation types in somatic cells. Deciphering mutational signatures in cancer provides insight into the mechanisms of cancer progression and this comprehensive genome analysis enables researchers to not only learn the current status of cancer predisposing genes but potentially to predict their future behavior through the understanding of the molecular underpinnings from which they arose.

The advancement of molecular techniques and gene sequencing platforms has enabled the discovery of additional genes, beyond *BRCA1* and *BRCA2*, that play a role in the development of hereditary breast and ovarian cancers. In a review of *PALB2*, a functional protein partner of *BRCA2*, Wu et al. discuss its function and role in breast cancer (5). Patients with monoallelic *PALB2* mutations are susceptible to breast, pancreatic, and ovarian cancer. *PALB2* mutation carriers are predisposed to breast cancer with a similar cumulative risk as *BRCA2* and having as much as a nine-fold higher than average lifetime risk, particularly in males. Emphasizing that *PALB2*-mutated breast cancers are associated with aggressive clinicopathologic features and poor prognosis, the authors recommend the inclusion of *PALB2* in multigene panels. Importantly, they introduce the possibility of effectiveness of poly (ADP-ribose) polymerase (PARP) inhibitors for *PALB2*-deficient breast tumors.

PARP is essential in the repair of single-stranded DNA breaks by homologous recombination (HR). However, in HR-deficient tumors PARP inhibitors prevent DNA repair *via* synthetic lethality. In their meta-analysis, Wang et al. assessed the efficacy of PARP inhibitors in newly diagnosed advanced stage ovarian cancers (6). Analysis of three randomized controlled trials revealed that maintenance therapy by PARP inhibitors improved progression free survival when compared to placebo, with only minimal adverse events. These findings were also confirmed on subgroup analysis, which showed improved survival regardless of age and stage at diagnosis, especially in patients with HR-deficiency and *BRCA* mutations.

The relationship between PARP inhibitor efficacy and *BRCA* mutations was further investigated in original research by Peixoto et al. who sought to determine the frequency of somatic and germline *BRCA* mutations in non-mucinous ovarian cancers, with focus on those with Portuguese ancestry (7). They discovered pathogenic variants in 19.3% of patients (13.3% germline, 5.9% somatic), with higher prevalence in tumors with high-grade serous morphology. In addition, they determined that identification of the most common deleterious variants in their population would be the most efficacious strategy for early detection and management.

In addition to PARP inhibitors, HR-deficient tumor cells can be sensitive to platinum compounds. In their mini-review, Pouptsis et al. describe the most recent systemic treatment advances and clinical outcomes of hereditary breast cancer patients treated with platinum-based regimens and PARP inhibitors (8). In addition, they discuss risk-reducing surgical management options and challenges associated with such interventions in young patients.

HBOC may be suspected in different clinical scenarios, including cancer diagnosis before the age of 50 years or in multiple first and/or second degree relatives on the same side of the family, diagnosis of second ipsilateral or contralateral breast cancer or both breast and ovarian cancers, diagnosis of breast cancer in a male relative, or cancer history in a family of Ashkenazi Jewish ancestry. However, approximately one-third of HBOC patients would not qualify for germline mutation testing based on family history alone. One effective way to triage patients for genetic screening is through microscopic examination of their tumors. A perspective article by Hodgson and Turashvili describe the unique pathologic features of *BRCA*-associated breast and ovarian carcinomas (9). They contrast the *BRCA1* mutated breast cancers which are frequently high-grade and triple-negative with medullary morphology with those of *BRCA2* carriers which are more similar to sporadic ER-positive luminal-type tumors. *BRCA*-associated ovarian tumors are almost exclusively high-grade serous carcinomas, often exhibiting the so-called “SET (Solid, pseudo-Endometrioid, and Transitional cell carcinoma-like) features.” They emphasize the importance of accurate pathologic assessment to ensure that patients receive optimal management, including genetic screening.

As screening programs, genetic testing and preventative measures have demonstrated to reduce HBOC-related mortality by half since the discovery of the *BRCA* genes and their role in HBOC, the data presented in this Research Topic will hopefully serve as an important reminder to clinicians, pathologists, geneticists, and other medical professionals as well as trainees that a multidisciplinary approach is critical in order to help *BRCA* mutation carriers make informed decisions regarding the screening, prevention, and treatment of hereditary breast and ovarian cancer.

## Author Contributions

All authors listed have made a substantial, direct and intellectual contribution to the work, and approved it for publication.

## Conflict of Interest

The authors declare that the research was conducted in the absence of any commercial or financial relationships that could be construed as a potential conflict of interest.
